# Supplementary material for: Structure of the human epithelial sodium channel by cryo-electron microscopy
Source: eLife. 2018 Sep 25;7:e39340. doi: 10.7554/eLife.39340 (PMC6197857; doi:10.7554/eLife.39340)
Supplement: Figure 2—source data 1. [file elife-39340-fig2-data1.docx]

**Figure 2** – source data 1

|  | **FL-ENaC** | **Δα-FLβγ** | **Δα*-FLβγ** | **Δβ-FLαγ** | **Δγ-FLαβ** | **Δγ*-FLαβ** |
| --- | --- | --- | --- | --- | --- | --- |
|  |  |  |  |  |  |  |
| Oocyte 1 | 2.78 | 5.69 | 4.06 | 1.36 | 12.29 | 6.71 |
| Oocyte 2 | 2 | 5.91 | 4.08 | 1.48 | 6.55 | 16.34 |
| Oocyte 3 | 1.88 | 3.85 | 5.12 | 1.55 | 9.73 | 16.72 |
|  |  |  |  |  |  |  |
| Mean | 2.22 | 5.15 | 4.41 | 1.46 | 9.52 | 13.26 |
| Standard Deviation | 0.49 | 1.13 | 0.61 | 0.1 | 2.88 | 5.67 |

Note: Ratio of measured steady state currents pre- and post trypsin treatment (I_Post_/I_Pre_, 2.5 µg/mL for 5 min).
