# Supplementary material for: Structure of the human epithelial sodium channel by cryo-electron microscopy
Source: eLife. 2018 Sep 25;7:e39340. doi: 10.7554/eLife.39340 (PMC6197857; doi:10.7554/eLife.39340)
Supplement: Figure 3—source data 1. [file elife-39340-fig3-data1.docx]

| **F****igure 3 -- source data 1. Statistics of data collection, three-dimensional reconstruction and model refinement** | | | | | |
| --- | --- | --- | --- | --- | --- |
|  |  |  | |  |  |
|  | ∆ENaC-10D4_−_1 | ∆ENaC-10D4_−_2 | | ∆ENaC-7B1/10D4_−_1 | ∆ENaC-7B1/10D4_−_2 |
| **Fab** | 10D4 | 10D4 | | 7B1, 10D4 | 7B1, 10D4 |
| **Data collection** |  |  | |  |  |
| Microscope | FEI Krios | FEI Krios | | FEI Krios | FEI Krios |
| Voltage (kV) | 300 | 300 | | 300 | 300 |
| Detector | Gatan K2 Summit | Gatan K2 Summit | | Gatan K2 Summit | Gatan K2 Summit |
| Detector mode | Super resolution | Counting | | Super resolution | Super resolution |
| Defocus range (µm) | −1.5 - −3.5 | −1.5 - −3.0 | | −1.0 - −2.5 | −0.8 - −1.75 |
| Exposure time (s) | 15 | 15 | | 10 | 12 |
| Dose rate (e^−^/Å^2^/s) | 3.7 | 3.3 | | 6.2 | 6 |
| Number of frames | 30 | 60 | | 40 | 48 |
| Pixel Size (Å) | 1.33 | 1.33 | | 1.33 | 1.33 |
| Total Dose (e^−^/Å^2^) | 54 | 50 | | 62 | 71 |
|  |  |  | |  |  |
| **Data processing** |  |  | |  |  |
| Motion correction | UCSF MotionCor2 | UCSF MotionCor2 | | UCSF MotionCor2 | UCSF MotionCor2 |
| CTF estimation | Gctf | Gctf | | Gctf | Gctf |
| Particle Picking | DoG Picker | DoG Picker | | DoG Picker/Manually | DoG Picker/Manually |
| 2D/3D classification | Relion2.0 | Relion2.0 | | Relion2.0 | Relion2.0 |
| 3D class/refinement | Relion2.0/CryoSPARC | Relion2.0/CryoSPARC | | CryoSPARC/Frealign | CryoSPARC/Frealign |
|  |  | |  | | |
| **Reconstruction** | **CryoSPARC** | | **CryoSPARC/Frealign** | | |
| Symmetry | C1 | | C1 | | |
| Particles processed | 41,459 | | 329,180 | | |
| Particles refined | 41,459 | | 244,223/290,007 | | |
| Resolution masked (Å) | 5.4 | | 4.2/4.2 | | |
| Map sharpening |  | | -200 | | |
| B-factor (Å^2^) |  | |  |  |  |
| **Reconstruction final map** | |  | | **Frealign** | |
| Symmetry |  |  | | C1 | |
| Particles refined |  |  | | 302,263 | |
| Resolution masked (Å) |  |  | | 3.9 | |
|  |  |  | |  |  |
| **Model statistics** |  |  | |  |  |
| Protein residues |  |  | | 1813 | |
| Resolution (FSC=0.143, Å) | |  | | 4.0 | |
| Molprobity score |  |  | | 5 | |
| Cß deviations |  |  | | 0 | |
| Ramachandran |  |  | |  |  |
| Outliers |  |  | | 0 | |
| Allowed |  |  | | 4% | |
| Favored |  |  | | 96% | |
| RMS deviations |  |  | |  |  |
| Bond length |  |  | | 0.003 | |
| Bond angles |  |  | | 0.656 | |
