# Supplementary material for: Structure of the human epithelial sodium channel by cryo-electron microscopy
Source: eLife. 2018 Sep 25;7:e39340. doi: 10.7554/eLife.39340 (PMC6197857; doi:10.7554/eLife.39340)
Supplement: Transparent reporting form [file elife-39340-transrepform.pdf]

eLIFE
1st Floor
24 Hills Road
Cambridge CB2 1JP, UK
P 01223 855340
W elifesciences.org
T @elife

eLife's transparent reporting form

We encourage authors to provide detailed information *within their submission* to facilitate the interpretation and replication of experiments. Authors can upload supporting documentation to indicate the use of appropriate reporting guidelines for health-related research (see EQUATOR Network), life science research (see the BioSharing Information Resource), or the ARRIVE guidelines for reporting work involving animal research. Where applicable, authors should refer to any relevant reporting standards documents in this form.

If you have any questions, please consult our Journal Policies and/or contact us: editorial@elifesciences.org.

Sample-size estimation

- You should state whether an appropriate sample size was computed when the study was being designed
- You should state the statistical method of sample size computation and any required assumptions
- If no explicit power analysis was used, you should describe how you decided what sample (replicate) size (number) to use

Please outline where this information can be found within the submission (e.g., sections or figure legends), or explain why this information doesn't apply to your submission:

No power analysis was used to determine sample size. Sample size for cryo-EM experiments was determined based on access to the Krios microscope at the Multiscale Microscopy Core (OHSU). For two-electrode voltage clamp electrophysiology experiments, sample size was dependent on the number of oocytes that were in optimal condition for RNA injection, as well as reproducibility of the recordings. Conditions were based on color and shape of the oocytes. The sample size for the cryo-EM experiments is in Figure 3 –figure supplement 1. The sample size for the TEVC experiments can be found in legends of Figure 2, and Figure 2 – figure supplement 3.

Replicates

- You should report how often each experiment was performed
- You should include a definition of biological versus technical replication
- The data obtained should be provided and sufficient information should be provided to indicate the number of independent biological and/or technical replicates
- If you encountered any outliers, you should describe how these were handled
- Criteria for exclusion/inclusion of data should be clearly stated
- High-throughput sequence data should be uploaded before submission, with a private link for reviewers provided (these are available from both GEO and ArrayExpress)

Please outline where this information can be found within the submission (e.g., sections or figure legends), or explain why this information doesn't apply to your submission:

eLife Sciences Publications, Ltd is a limited liability non-profit non-stock corporation incorporated in the State of Delaware, USA, with company number 5030732, and is registered in the UK with company number FC030576 and branch number BR015634 at the address 1st Floor, 24 Hills Road, Cambridge CB2 1JP | August 2014

For cryo-EM experiments, all 4 of the experiments were biological replications. Biological replicates are defined as protein obtained following independent infections of HEK293S cells. Micrographs with ice contamination and aggregates were excluded from particle picking. At the 3D classification stage, classes that gave rise to maps that resemble a channel with Fabs bound were selected for final reconstruction and refinement. Details are provided in the cryo-EM flowchart in Figure 3 – figure supplement 1.
For electrophysiology experiments, discolored oocytes post-RNA injections were excluded from the experiments.

eLife Sciences Publications, Ltd is a limited liability non-profit non-stock corporation incorporated in the State of Delaware, USA, with company number 5030732, and is registered in the UK with company number FC030576 and branch number BR015634 at the address 1st Floor, 24 Hills Road, Cambridge CB2 1JP | August 2014

Statistical reporting

- Statistical analysis methods should be described and justified
- Raw data should be presented in figures whenever informative to do so (typically when N per group is less than 10)
- For each experiment, you should identify the statistical tests used, exact values of N, definitions of center, methods of multiple test correction, and dispersion and precision measures (e.g., mean, median, SD, SEM, confidence intervals; and, for the major substantive results, a measure of effect size (e.g., Pearson's r, Cohen's d)
- Report exact p-values wherever possible alongside the summary statistics and 95% confidence intervals. These should be reported for all key questions and not only when the p-value is less than 0.05.

Please outline where this information can be found within the submission (e.g., sections or figure legends), or explain why this information doesn't apply to your submission:

No statistical tests were used.

(For large datasets, or papers with a very large number of statistical tests, you may upload a single table file with tests, Ns, etc., with reference to sections in the manuscript.)

Group allocation

- Indicate how samples were allocated into experimental groups (in the case of clinical studies, please specify allocation to treatment method); if randomization was used, please also state if restricted randomization was applied
- Indicate if masking was used during group allocation, data collection and/or data analysis

Please outline where this information can be found within the submission (e.g., sections or figure legends), or explain why this information doesn't apply to your submission:

For Cryo-EM experiments, particles in micrographs were picked using the program DoGPicker, and Relion was used for 2D and 3D classification to remove ice contamination, micelles and denatured protein. For structure refinement using cryoSPARC, particles were split into two groups to generate half maps and then used to calculate gold standard FSC. More details provided in the Methods section.
For Electrophysiology experiments, we characterized ion channel properties with different subunit composition/mutant combination. We measured activity of oocytes that demonstrated currents blocked by amiloride. Details provided in pages 7 & 8, legends of Figure 2, Figure 2 – figure supplement 1, and Figure 2 – figure supplement 2. We have also provided numerical data as a source data corresponding to the graph shown in Figure 2 – figure supplement 3

Additional data files ("source data")

- We encourage files, such as numerical data that are represented as a graph in you to upload relevant additional data a figure, or as a summary table
- Where provided, these should be in the most useful format, and they can be uploaded as "Source data" files linked to a main figure or table
- Include model definition files including the full list of parameters used
- Include code used for data analysis (e.g., R, MatLab)
- Avoid stating that data files are "available upon request"

Please indicate the figures or tables for which source data files have been provided:

The three-dimensional cryo-EM density map and the coordinate for the structure of ΔENAC have been deposited in the EM Database and Protein Data Bank under the accession codes EMD-7130 and 6BQN, respectively.
